# Inhibitory Effect of Corn Silk on Skin Pigmentation

**DOI:** 10.3390/molecules19032808

**Published:** 2014-03-03

**Authors:** Sang Yoon Choi, Yeonmi Lee, Sung Soo Kim, Hyun Min Ju, Ji Hwoon Baek, Chul-Soo Park, Dong-Hyuk Lee

**Affiliations:** 1 Korea Food Research Institute, Seongnam 463-746, Korea; E-Mails: sweetlinda@naver.com (Y.L.); sung@kfri.re.kr (S.S.K.); yskx24@naver.com (H.M.J.); 2 Department of Food Biotechnology, University of Science and Technology (UST), Daejeon 305-350, Korea; 3 Dermapro Co. Ltd., Seoul 156-709, Korea; E-Mail: dermapro@dermapro.co.kr; 4 Kwang-Dong Pharmaceutical Co. Ltd, Seoul 137-875, Korea; E-Mails: parkcs@ekdp.com (C.-S.P.); dhlee@ekdp.com (D.-H.L.)

**Keywords:** corn silk, melanin, skin, tyrosinase

## Abstract

In this study, the inhibitory effect of corn silk on melanin production was evaluated. This study was performed to investigate the inhibitory effect of corn silk on melanin production in Melan-A cells by measuring melanin production and protein expression. The corn silk extract applied on Melan-A cells at a concentration of 100 ppm decreased melanin production by 37.2% without cytotoxicity. This was a better result than arbutin, a positive whitening agent, which exhibited a 26.8% melanin production inhibitory effect at the same concentration. The corn silk extract did not suppress tyrosinase activity but greatly reduced the expression of tyrosinase in Melan-A cells. In addition, corn silk extract was applied to the human face with hyperpigmentation, and skin color was measured to examine the degree of skin pigment reduction. The application of corn silk extract on faces with hyperpigmentation significantly reduced skin pigmentation without abnormal reactions. Based on the results above, corn silk has good prospects for use as a material for suppressing skin pigmentation.

## 1. Introduction

The skin color of the human body is determined by melanin, carotenoids, hemoglobin, and bilirubin, among which melanin is the most important factor [1,2]. Melanin is produced to protect the skin against damage due to UV radiation [3]. The biosynthesis of melanin starts with the oxidation of tyrosine by tyrosinase; DOPA and dopachrome are then produced, followed by DHI-eumelanin, DHICA-eumelanin, and pheomelanin [4,5]. Human skin is made of three layers: the epidermis, dermis, and subcutaneous tissue. Of these the epidermis is the outermost layer, where melanocytes that produce melanin exist in the basal lamina or the lower part of the epidermis [6]. Skin darkening occurs when the melanin produced by the melanin-producing cell (melanocyte) is transferred to the keratinocyte and accumulates in the epidermis.

Although melanin protects the skin, the hyper-production of melanin pigment can cause melasma, freckles, and dark spots [7,8]. To prevent or improve skin darkening due to melanin hyper-production, skin pigment-suppressing agents using kojic acid, arbutin, or licorice extract have been developed, but various problems such as adverse side effects and weak efficacy have been observed [9]. Thus, developing a new skin pigment-inhibiting agent is a matter of urgency.

Corn silk is the thin, yellow- or brown-colored thread-like styles at the tip of an ear of corn (*Zea mays* L.), an annual plant of the Gramineae family containing various flavonoids and terpenoids [10]. As for its physiological activities, corn has been reported to have antioxidant [11], anti-inflammatory [12], anti-diabetic [13], anti-fatigue [14], and neuroprotective effects [15]. In particular, its diuretic action is well known [16]. However, the activity of corn silk related to skin pigment suppression has yet to be reported. Thus, the study was performed to investigate the inhibitory effect of corn silk on melanin production in Melan-A cells by measuring melanin production and protein expression. Melan-A cells are highly pigmented melanocytes and provide an excellent parallel non-tumorigenic cell line derived from C57Bl/6 mice [17]. In addition, corn silk extract aqueous solutions were applied on the human face with hyperpigmentation twice a day for 8 weeks, and skin color was measured to check for any adverse reactions and examine the degree of skin pigment reduction.

## 2. Results and Discussion

### 2.1. Effects on Cell Viability and Melanin Production

**Figure 1:**
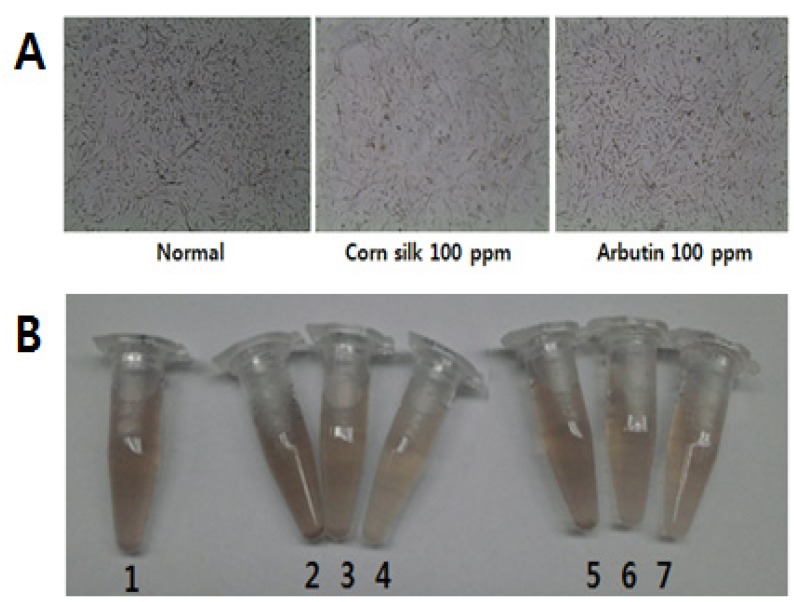
Effects of corn silk extract and arbutin on Melan-A cells. (**A**) Melan-A cell image after 3 days’ corn silk treatment (magnification 200×); (**B**) Effects of corn silk extract on the color of a Melan-A cell pellet.

The results of cell viability and melanin production after 3 days of sample treatment on Melan-A cells showed that corn silk extract at the 100 ppm concentration decreased melanin production by 37.2% without cytotoxicity. This was a 10.4% more decrease in melanin than achieved with arbutin, one of the positive depigmenting agents [18], which exhibited 26.8% melanin production inhibitory effect at the same concentration (Figure 1 and Table 1). Changes in cell images and color of cell pellets by concentration after 3 days of corn silk extract or arbutin treatment are shown in Figure 1.

### 2.2. Tyrosinase Inhibitory Effect

**Figure 2:**
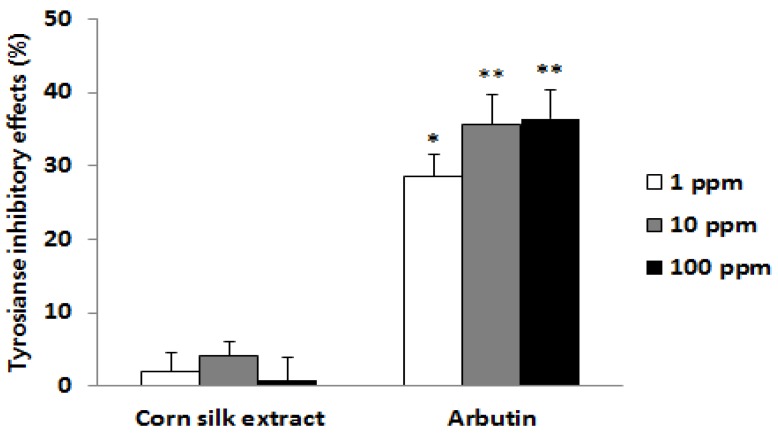
Tyrosinase inhibitory effects.

Tyrosinase is an important enzyme that stimulates the oxidation of tyrosine and L-dopa at the early stage of melanin biosynthesis. Thus, the search for a tyrosinase inhibitor has been a major target of research in the development of skin pigment-inhibiting agents [19]. To find out whether corn silk extract inhibits tyrosinase activity or not, tyrosinase and its substrate L-dopa were incubated with corn silk extract; the amount of dopachrome produced was then measured using the absorbance. Results showed that the activity of tyrosinase was not inhibited at all concentrations (Figure 2). Therefore, the inhibitory effect of corn silk extract on melanin production was believed to involve another mechanism in addition to the inhibition of tyrosinase activity.

1: No treatment, 2: Corn silk 1 ppm, 3: Corn silk 10 ppm, 4: Corn silk 100 ppm, 5: Arbutin 1 ppm, 6: Arbutin 10 ppm, 7: Arbutin 100 ppm.

**Table 1:** Effects on cell viability and melanin production in Melan-A cells.

	Concentrations (ppm)	Cell viability (%)	Melanin production (%)
Corn silk	1	97.5 ± 1.7	100.0 ± 0.8
	10	95.0 ± 4.2	90.8 ± 4.1
	100	96.9 ± 4.9	62.8 ± 4.9
Arbutin	1	97.1 ± 3.3	99.7 ± 2.6
	10	95.7 ± 3.6	99.4 ± 5.0
	100	100.8 ± 2.7	73.2 ± 4.8

Viability and melanin content of vehicle were set to 100%. Data represent the mean ± SD of triplicate experiments.

Data represent the mean of triplicate experiments (mean ± SEM, * *p* < 0.05, ** *p* < 0.01).

### 2.3. Effect on Protein Expression in the Cell

**Figure 3:**
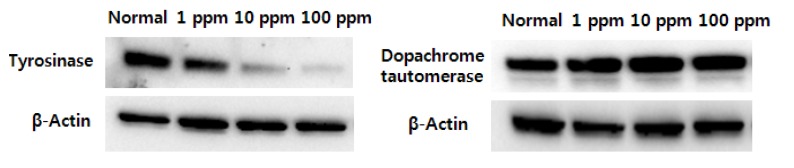
Effects of corn silk extract on the expression of tyrosinase and dopachrome tautomerase (TRP-2).

Melan-A cells were treated with corn silk extract for 3 days, and proteins were then extracted for the quantification of tyrosinase and TRP-2 expression, which are important enzymes in melanin biosynthesis, using western immunoblotting; as a result, corn silk extract reduced tyrosinase expression in a concentration-dependent manner but did not affect TRP-2 expression at any of the concentrations (Figure 3). TRP-2 (dopachrome tautomerase) has been known to play a major role, along with tyrosinase, in the biosynthesis of melanin. Specifically, TRP-2 plays a role in the conversion of dopachrome into dihydroxyindole-2-carboxylic acid, and finally into DHICA-eumelanin [20,21]. Thus, corn silk extract is considered to reduce melanin production not by regulating TRP-2, which is involved in the middle stage of melanin biosynthesis, but by minimizing the expression of tyrosinase in the cells involved in the early stages of melanin biosynthesis pathways.

### 2.4. Improvement in Hyperpigmentation on Human Skin

**Figure 4:**
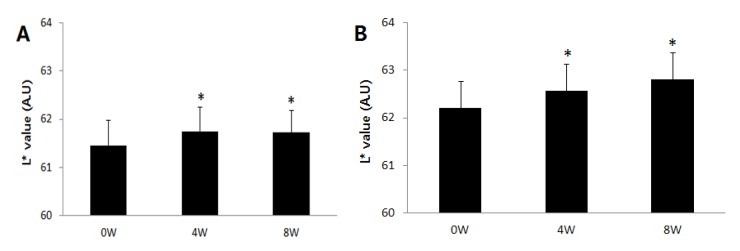
Changes in skin brightness (L* value) following 8 consecutive weeks’ use of corn silk extract (mean ± SEM, * *p* < 0.05 *vs.* before treatment). (**A**) corn silk extract 0.75% aqueous solution; (**B**) corn silk extract 1.5% aqueous solution.

**Figure 5:**
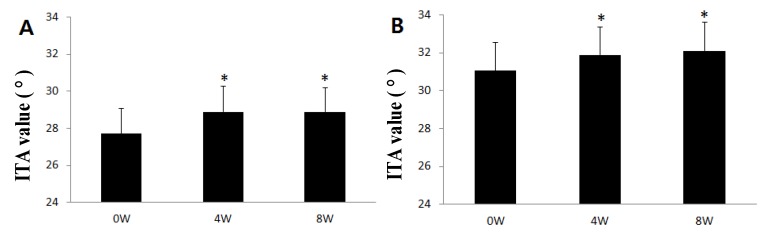
Changes in skin color (ITA value) following 8 consecutive weeks’ use of corn silk extract (mean ± SEM, * *p* < 0.05 *vs.* before treatment). (**A**) corn silk extract 0.75% aqueous solution; (**B**) corn silk extract 1.5% aqueous solution.

**Figure 6:**
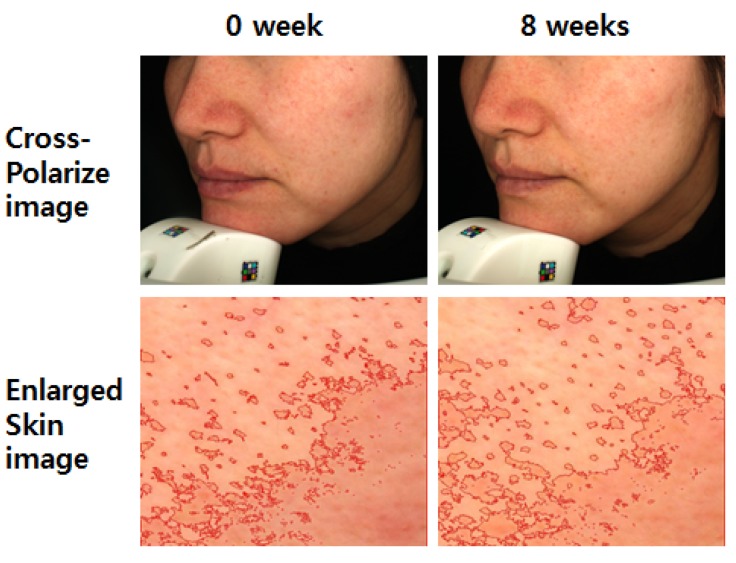
Skin color images following 8 consecutive weeks’ use of corn silk extract 1.5% aqueous solution.

**Figure 7:**
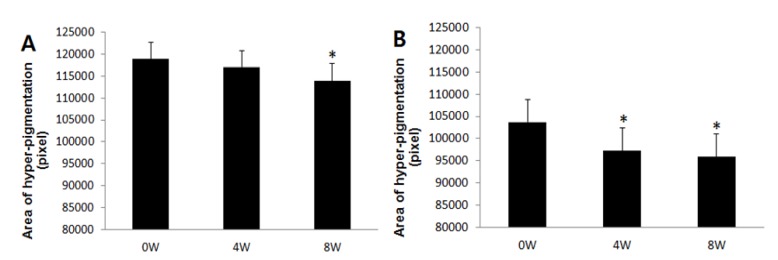
Changes in hyperpigmentation area following 8 consecutive weeks’ use of corn silk extract (mean ± SEM, * *p* < 0.05 *vs.* before treatment). (**A**) corn silk extract 0.75% aqueous solution; (**B**) corn silk extract 1.5% aqueous solution.

**Figure 8:**
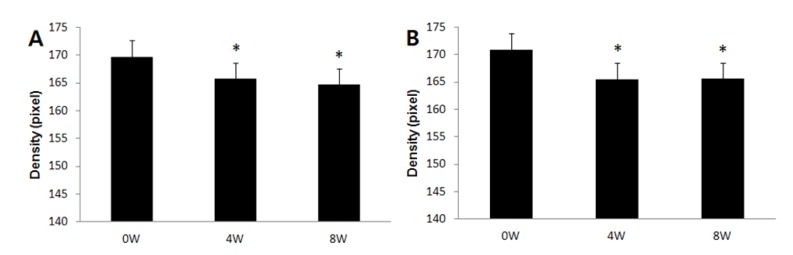
Changes in hyperpigmentation density following 8 consecutive weeks’ use of corn silk extract (mean ± SEM, * *p* < 0.05 *vs.* before treatment). (**A**) corn silk extract 0.75% aqueous solution; (**B**) corn silk extract 1.5% aqueous solution.

According to skin color analysis results using a spectrophotometer, both corn silk extract 0.75% aqueous solution and 1.5% aqueous solution significantly increased the L* value and ITA value after 4 weeks and 8 weeks of application compared to before the application (*p* < 0.05, Figure 4 and Figure 5). Moreover, the skin color image of the subjects was captured for the analysis of the area and density of hyperpigmentation. The representative skin color images of the subjects using corn silk extract 1.5% aqueous solution after 8 weeks of use are shown in Figure 6. The results of the analysis of the area and density of hyperpigmentation using an image analysis program were as follows: in the case of the corn silk extract 0.75% aqueous solution, the area of hyperpigmentation significantly decreased after 8 weeks of use, and the density of hyperpigmentation decreased after 4 weeks and 8 weeks of use, compared to before use. In the case of corn silk extract 1.5% aqueous solution, both area and density of hyperpigmentation significantly decreased after 4 weeks and 8 weeks of use (Figure 7 and Figure 8).

### 2.5. Safety on Human Skin

Abnormal skin reactions and stimulation were not observed in any of the subjects during 8 consecutive weeks of use of corn silk extract aqueous solution (Table 2).

**Table 2:** Adverse skin reactions (*n* = 21 for each group, number of subjects).

	Symptom	Corn silk extract 0.75%		Corn silk extract 1.5%	
		4W	8W	4W	8W
Irritation	Redness	0	0	0	
	Edema	0	0	0	0
	Desquamation	0	0	0	0
	Papule	0	0	0	0
	Other	0	0	0	0

### 2.6. Discussion

Despite its diuretic, antioxidant and anti-inflammatory effects, corn silk has been mostly discarded rather than utilized, being used only as part of the ingredients for food additives and corn silk tea. In this study, corn silk extract demonstrated excellent melanin production inhibitory effect on Melan-A cells without cytotoxicity. Such inhibitory activity on melanin was higher at the same concentration when compared to arbutin, a single substance with known skin whitening functions. In fact, the corn silk extract greatly suppressed tyrosinase expression in the cells. Thus, the inhibitory effect of corn silk on melanin production is believed to be due to the reduction of tyrosinase expression in the cell. Our future studies will investigate its effect on upper regulators including MITF and p-CREB. Moreover, female subjects with hyperpigmentation on the face showed improvement in skin color without abnormal reactions after the application of corn silk extract 0.75% and 1.5% solutions twice a day for 8 weeks. Therefore, corn silk is deemed to have high potential as a skin whitening material. In addition, the luteolin content of the prepared corn silk extract was determined for standardization. Although a recent study reported that leteolin exhibited depigmenting effects in melanoma cell [22], the concentration of luteolin in the prepared corn silk extract was low (38.9 ppm). Therefore, the development of a new skin whitening substance from corn silk is expected to follow suit in the future study, based on the results of this study. The effect of corn silk in relation to melanin inhibition has yet to be reported. The biggest significance of this study are the inhibitory effects and mechanism of action of corn silk on melanin production in melanin-producing cells and the depigmenting activity in human skin that was tested, and the possibility of corn silk being used as skin pigment-inhibiting material being reported for the first time.

## 3. Experimental

### 3.1. Materials

L-Dopa, arbutin and 3-(4,5-dimethylthiazol-2-yl)-2,5-diphenyltetrazolium (MTT) were purchased from Sigma-Aldrich Co. (St. Louis, MO, USA). All solvents used for isolation were analytical grade products acquired from Merck Co. (Darmstadt, Germany). FBS (Fetal bovine serum), RPMI medium, and PS (Penicillin-Streptomycin) were purchased from Gibco BRL (Grand Island, NY, USA). Tyrosianse and TRP-2 antibody were purchased from Santa Cruz Biotech (Santa Cruz, CA, USA).

### 3.2. Extract Preparation

**Figure 9:**
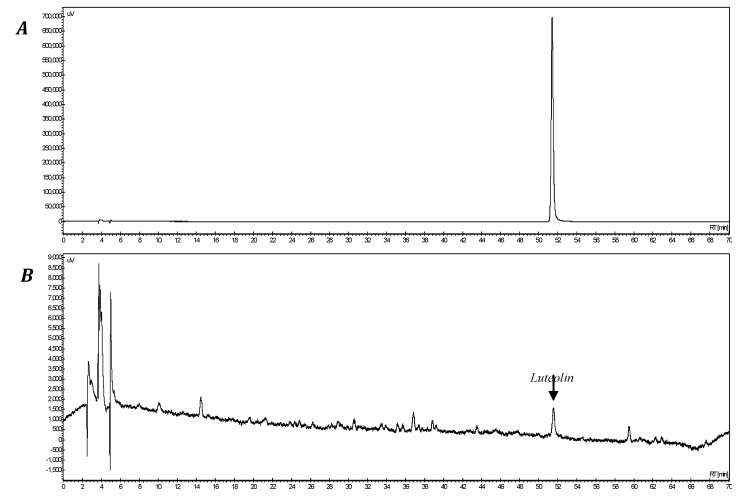
HPLC chromatogram of corn silk extract. (**A**) luteolin, (**B**) corn silk extract.

Corn silk was collected from Jilin Province, China in 2012. It was washed with purified water and sterilized at 135 °C for 60 min and extracted by adding purified water 25 times at 100 °C for 90 min. After filtration, it was vacuum-evaporated at 60 °C to produce corn silk extract. The content of luteolin was quantitatively analyzed with an HPLC system (Jasco, Tokyo, Japan) under the following conditions: using a Sunfire C18 column (5 µm, 4.6 × 250 mm), 2% acetic acid in water (solvent A) and 0.5% acetic acid in 50% acetonitrile (solvent B) were mixed in 90% solvent A in the beginning of the analysis, then after 60 min of the analysis, the solution was switched to a 20% solvent A using a mobile phase gradient. The flow was maintained at 0.8 mL/min and the absorbance were measured at 330 nm. As the results, luteolin was eluted at 51.6 min (Figure 9). The luteolin content of the prepared corn silk extract was 38.9 ppm.

### 3.3. Cell Culture

Melan-A cells, mouse melanocytes [17], were cultured at 37 °C, 5% CO_2_ using RPMI 1640 medium containing 10% fetal bovine serum, 1% penicillin-streptomycin, and 200 nM phorbol-12 myristate 13-acetate. Cells were inoculated on a 24-well plate at a concentration of 1 × 10^5^ cells/well and incubated for 24 h, and then treated with corn silk extract and arbutin for 3 days and incubated for another 24 h.

### 3.4. Cell Viability

After the elimination of the culture medium, cells were washed with PBS and then 200 μL of a crystal violet solution (0.1% crystal violet, 10% EtOH, the rest is PBS, *w/w*) was added to each well. Cells were incubated for 5 min at room temperature, and then washed with distilled water twice, and 1 mL of EtOH was added. After shaking for 10 min at room temperature, absorbance was measured at 590 nm.

### 3.5. Melanin Production

After the elimination of the culture medium, cells were washed with PBS, and 1 mL of 1 N NaOH was added to each well to dissolve the melanin. Absorbance was then measured at 400 nm.

### 3.6. Tyrosinase Inhibitory Effect

L-Dopa (120 μL, 8.0 mM) dissolved in 67 mM phosphate buffer (pH 6.8) and sample (40 μL) dissolved in methanol were placed in a 96-well microplate, and mushroom tyrosinase (40 μL, 125 U/mL) was then added. After incubation at 37 °C for 20 min, the amount of dopachrome produced was measured at 492 nm.

### 3.7. Protein Expression Level in the Cell

Melan-A cells (5 × 10^5^) were inoculated onto a culture dish and incubated for 24 h, and then treated with the samples for 3 days. Cells were incubated for another 24 h and subsequently collected, and lysis buffer (50 mM Tris-HCl, pH 8.0, 0.1% SDS, 150 mM NaCl, 1% NP-40, 0.02% sodium azide, 10 μg/mL PMSF, 1 μg/mL aprotinin) was added and the mixture sonicated to extract the proteins inside the cells. After electrophoresis of 50 μg of extracted protein using 8% SDS-polyacrylamide gel, it was transferred onto the membrane and blocked with 5% skim milk. The membrane was made to react with tyrosinase and dopachrome tautomerase (TRP-2) primary antibody followed by anti-goat secondary antibody and was detected using enhanced chemiluminescence detection (Bio-Rad Laboratories, Hercules, CA, USA).

### 3.8. Improvement of Hyperpigmentation in Human Skin

The subjects were 42 females aged 36–50 years (average: 42.9 ± 3.9) with hyperpigmentation on the face. The subjects were randomly divided into two groups and treated with either corn silk extract 0.75% aqueous solution or 1.5% aqueous solution (*w/w*) for 8 weeks twice a day (morning and evening) by applying all over the face after washing. Skin color was evaluated before use and at 4 weeks and 8 weeks after use by skin color measurement, and area and density of hyperpigmentation in subjects who washed their face and waited for 20 min at constant temperature and humidity (22 ± 2 °C, 50 ± 5%). At each time point, skin color on the side of the face was measured using a spectrophotometer CM-2500d (Minolta, Tokyo, Japan) for the L* value and ITA value. The formula of ITA value is [Arc tangent ((L* − 50)/b*)] × 180/3.14159. Likewise, the area and density of hyperpigmentation were measured using a facial image capturing system, VISIA ver.5 (Canfield, Fairfield, NJ, USA), and an image analysis program called Image-pro plus (MediaCybernetics, Bethesda, MD, USA).

### 3.9. Safety Evaluation on Human Skin

For safety evaluation, the corn silk extract aqueous solution was applied to the skin at 4 weeks and 8 weeks after use; subjective irritation and objective irritation by the subject's observation were evaluated.

### 3.10. Statistical Analysis

The statistical significance of the data was analyzed using the SPSS Package Program (IBM, Armonk, NY, USA). For human experiments, the Shapiro-Wilks test was applied for the normality test, the comparison by time point used repeated measures ANOVA for the parametric test and Wilcoxon’s signed-ranks test followed by post hoc evaluation for the non-parametric test.

## 4. Conclusions

In conclusion, corn silk may act as a depigmenting material for suppressing skin hyper-pigmentation by reducing tyrosinase expression.
